# Percutaneous Ultrasound-Assisted Carpal Tunnel Release Using Sono-Instruments®

**DOI:** 10.7759/cureus.66899

**Published:** 2024-08-14

**Authors:** Fabian Moungondo, Mohammad O Boushnak, Frédéric Schuind

**Affiliations:** 1 Department of Orthopedics and Traumatology, Université Libre de Bruxelles, Erasme University Hospital, Brussels, BEL; 2 Department of Orthopedic Surgery, Joondalup Orthopedic Group, Joondalup Health Campus, Perth, AUS

**Keywords:** outpatient clinic, minimally invasive surgery, percutaneous surgery, sono-instrument®, percutaneous carpal tunnel release

## Abstract

Percutaneous ultrasound-assisted carpal tunnel (CT) release is an emerging minimally invasive technique in hand surgery that could reduce complications, enhance patient satisfaction, and facilitate earlier return to daily activities. Among the various devices employed for this procedure, the Sono-Instrument allows pin-hole surgery. Its safety and effectiveness have been established. This study presents the technical nuances, pearls and pitfalls, advantages, and challenges of using the Sono-Instrument for percutaneous ultrasound-assisted CT release.

## Introduction

Carpal tunnel (CT) syndrome is the most frequent compressive neuropathy [1,2]. When conservative treatment is insufficient to relieve the symptoms, surgical release of the transverse carpal ligament (TCL) is indicated, which can be open/mini-open, endoscopic, or sonography-guided [3]. Minimally invasive techniques were developed to alleviate postoperative pain and wound healing issues after classical open release and to allow earlier return to daily activities and work [4]. Mini-open and endoscopic techniques are less invasive than open release, but still involve a small incision and are associated with a higher risk of iatrogenic injury [5,6].

Several cadaveric and clinical studies have demonstrated the feasibility of sonography-guided percutaneous release of TCL, in addition to a low complication rate and high patient satisfaction [7-10]. Various devices have been developed for this purpose [7-10]. Knife-like instruments allow for antegrade or retrograde cutting of the TCL, after a small incision and tissue dissection to create the plane of insertion of the instrument [7-10]. Sectioning of TCL in a straight line using the aforementioned instruments exposes the patient to the risk of iatrogenic nerve injury in the case of anatomical abnormalities (thenar motor branch) [11-13], or in the case of excessive force during sectioning, damaging anatomical structures beyond the cut, such as the superficial palmar arch and the Berrettini medio-ulnar anastomosis [13-15]. Sharp hook knife may inadvertently cut flexor tendons and a large instrument introduced in the non-extensible osteofibrous tunnel could be responsible for acute nerve compression with post-operative median nerve neurapraxia [13]. The Sono-Instrument® (Spirecut AG, Muttenz, Switzerland) represents a recent interesting alternative [16,17]. The instrument is extremely small (1.5mm diameter), allowing the entire operation to be performed through a simple puncture (“pin-hole” procedure). The release is progressive, and the surgeon, guided by live-time ultrasound imaging, decides the safest direction and plane of TCL release and can change the orientation of the instrument, and consequently the sectioning plane at any time during the procedure, which could be safer than the straight cut of other instruments. The instrument, while effectively cutting tense TCL fibers, is not sharp enough to inadvertently injure less tense structures like tendons, vessels, or nerves [16,17]. Special features (lateral flange reflecting echoes at the cutting extremity, spiral groove on the rod) have been designed to enhance ultrasound visualization and also allow for rotatory orientation [16,17].

The aim of this article is to detail the technical nuances, pearls, and pitfalls of using the Sono-Instrument® for percutaneous TCL release.

## Technical report

Anatomy

A perfect understanding of the patient’s CT anatomy is of crucial importance to perform ultrasound-guided percutaneous release. Before the surgery, care is taken to identify in the palmar distal forearm and wrist the bony landmarks, the structures at risk, the TCL to be released, and the safe zone, located between the radial edge of the hamate hook and the median nerve. It is mandatory to check for unexpected disease or anatomical abnormality, like a distal radius malunion, a canalar ganglion, or any other peculiarity that could contraindicate the operation.

The important landmarks are the distal radius and pronator quadratus, the lunate, the hook of the hamate, the scaphoid and trapezial tubercles, the ulnar pedicle, and the TCL. Each structure at risk is then identified, particularly the superficial palmar arch, the ulnar and median nerves and their branches, and the flexor tendons. A dynamic evaluation of flexor tendon gliding is performed under sonography, by asking the patient to move the fingers, which helps in identifying tendons and median nerve. The surface of the median nerve, usually enlarged at the entrance of the CT, can be measured, and its intracanalar altered structure and area evaluated. The origin as well as the orientation of the motor branch is identified, and any abnormality in the origin and location of this nerve is ruled out. Distally, the Berrettini anastomosis between the median nerve and ulnar nerve is located accurately, to prevent any inadvertent iatrogenic laceration [14]. The surgeon finally determines the safe release zone, located ulnar to the median nerve and radial to the hook of the hamate, avoiding entering Guyon’s canal [7].

Indication and contraindications

Percutaneous CT release is indicated in symptomatic syndrome in an adult, confirmed by clinical signs, symptoms, and altered nerve conduction studies [1,2].

Contraindications to endoscopic release also apply for sonography-guided CT release, such as previous surgery, intracanal mass, anatomical abnormality including distal radius malunion, skeletal distortion, active infection, and altered coagulation (this is not an exhaustive list). Known allergies to local anesthetics and metals are other contraindications.

Surgical technique

The procedure starts by accurately identifying the structures contained inside the CT using sonography. The TCL is at a mean depth of 10 mm (8-12 mm) [18]. Modern equipment with a linear probe and B-mode MSK imaging is enough to proceed safely. Lower frequency might be indicated in a very thick wrist such as in a strong male manual worker. Excellent sonography imaging is in any case needed for precisely guiding the instrument in the exact, safe cutting plane after identification of anatomical structures at risk. In case of insufficient sonography visibility, converting to open release should be considered.

The operation is performed without a tourniquet or adrenalin, which allows good sonographic visualization of pulsating arteries. These arteries need to be preserved and represent important anatomical landmarks. As the surgery is percutaneous, and as the cut is made between thenar and hypothenar muscle insertions, in a relatively avascular zone, bleeding is minimal. In the infrequent occurrence of bleeding, the visibility of the anatomical structures and of the instrument is not altered; hence, a tourniquet or adrenaline injection is of no benefit.

**Figure 1:**
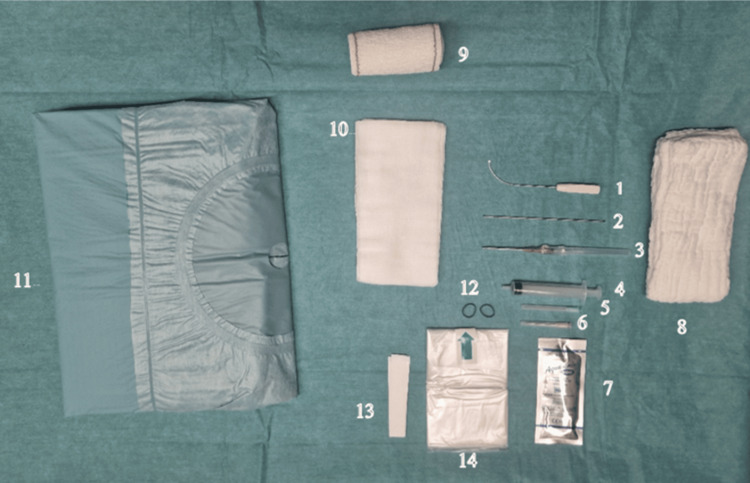
Material needed to perform the surgery. The probe cover (14) was attached to the probe with elastic rubber bands (12) and fixed on the sterile drape with tapes (13). A sterile ultrasound gel (7) is applied between the probe cover and the patient skin. The patient is positioned in the supine position with the operated hand placed on a table and stabilized with two abdominal sponges (8) in the neutral position of the wrist. An 18G needle (6) is used to harvest the lidocaine in a 10cc syringe (4) and a 20 G needle (5) for injection through the entry point. A 14G catheter (3) is then introduced through the skin and the forearm fascia to increase the entry point size and replaced by the round-ended stylus (2) to palpate the TCL and define the cut trajectory.  After the stylus removal, the cutting instrument (1) is introduced through the entry point to perform the antegrade release. After the release, the round-ended stylus is used to palpate the ligament and be sure of the completeness of its release. If this is not the case, the exact location of the remaining fibers is defined by probe palpation, and the cutting instrument is re-introduced to complete the release. Three 10x20 sponges (10) are then used to make a compressive dressing with a Velpeau band (9) that will be removed by the patient after 12 hours.

The required sterile material includes a hand drape, surgical gloves, the CT-Sono-Instrument, a rounded end stylus, a 10ml syringe, a 21-gauge 50 mm needle for local anesthesia, a 14-gauge intravenous catheter for skin and fascia entry point perforation to enter the CT, sterile sonographic gel, transparent cover for the sonographic probe, and sponges for the compressive dressing at the end of the operation. Support holding the hand may be useful; this might be as simple as a thick sponge. No other instruments are needed, such as hand surgery instrument sets (Figure 1).

The patient is comfortably placed in the supine position, with his/her arm resting on a lateral table (or the arms resting on lateral tables on both sides, in case of bilateral surgery). The surgeon sits close to the axilla or the shoulder of the patient, holding the instrument with his/her dominant hand, and the sonographic probe with his/her nondominant hand (therefore, the surgeon positions him/herself close to the head, or close to the belly of the patient, depending on the side operated and on the laterality of the operator). Note that the whole operation may be performed by a surgeon alone, without an assistant. Before proceeding, the setup of the patient and the sonography screen are evaluated to ensure comfort and ease of operation. Patient cooperation is assessed to confirm the feasibility of the operation under local anesthesia. Although the operation can be performed under regional or general anesthesia, local anesthesia is preferred. In addition to the advantage of lower morbidity, it is better suited for ambulatory surgery, and it allows potentially during the procedure the detection of dysesthesia, which would alert the operator to the proximity of nerves. In addition, the injection of anesthetics in the closed compartment of the CT radially displaces the median nerve, enlarging the safe zone (“hydro-dissection” phenomenon).

After the preoperative ultrasonographic evaluation, the limb is disinfected and draped in a sterile fashion. Before covering the sonographic probe with the sterile transparent cover, the gel is applied to the transducer, thereby preventing air from obstructing the ultrasound wave transmission.

**Figure 2:**
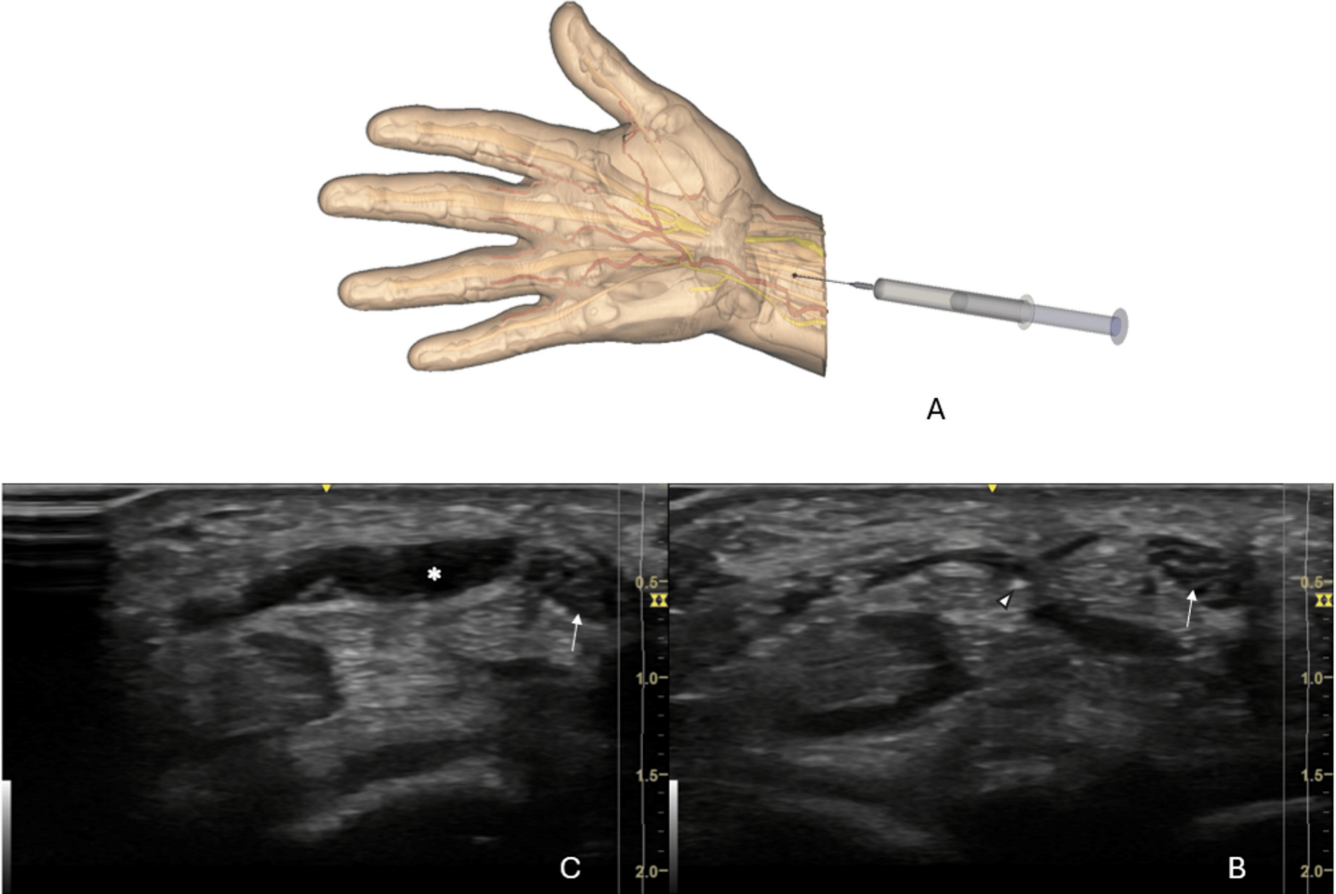
Location of the entry point (red dot) ~1 cm proximal to the wrist flexor crease. The first procedure step is antegrade lidocaine injection through this point (A). Sonographic short-axis view is used to control the needle positioning (arrowhead) relative to the median nerve (arrow) (B). During injection, the median nerve (arrow) is pushed by the fluid (star) to the radial side of the wrist (C). This figure is the original work of the authors.

The surgeon locates the safe zone and determines the optimal entry point, located about 1 cm proximal to the volar wrist crease, and ulnar to the Palmaris longus. The skin entry point is therefore proximal to the TCL, in the distal palmar forearm (Figure 2A). The first step is the injection of this entry point of local anesthetics (8ml lidocaine 1% without adrenalin), from proximal to distal, first in the CT, then superficially to the TCL. Care is taken to avoid injecting air, as it would interfere with sonographic imaging. The injection is moderately painful in the superficial plane. The injection usually allows transiently better visualize the TCL. During injection, the injected volume pushes the median nerve to the radial side of the wrist (Figures 2B, 2C).

**Figure 3:**
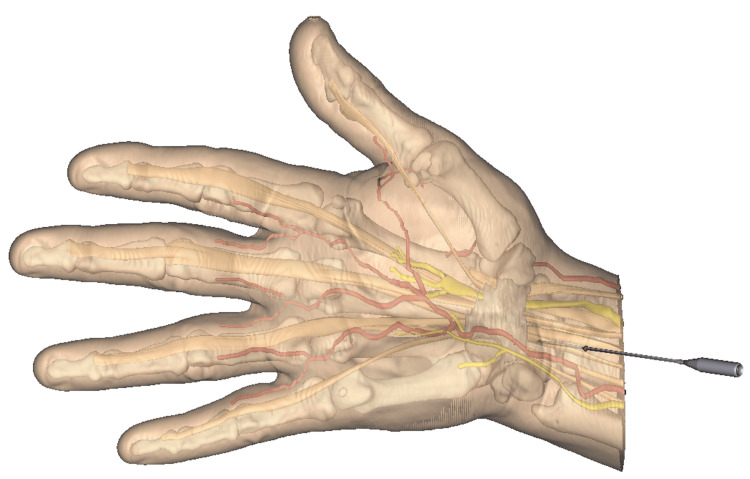
A 14-gauge catheter is introduced through the skin and fascia to enlarge the entry point in order to allow the introduction of the stylus and cutting instrument. This figure is the original work of the authors.

The next step is to make at the entry point a puncture skin and fascial opening, using the 14-gauge catheter (“pin-hole”). The tip of the catheter is accurately identified by doing slight rotations of its bevel. The catheter perforates the distal palmar antebrachial aponeurosis, making an inclination of about 35° in the sagittal plane (Figure 3).

**Figure 4:**
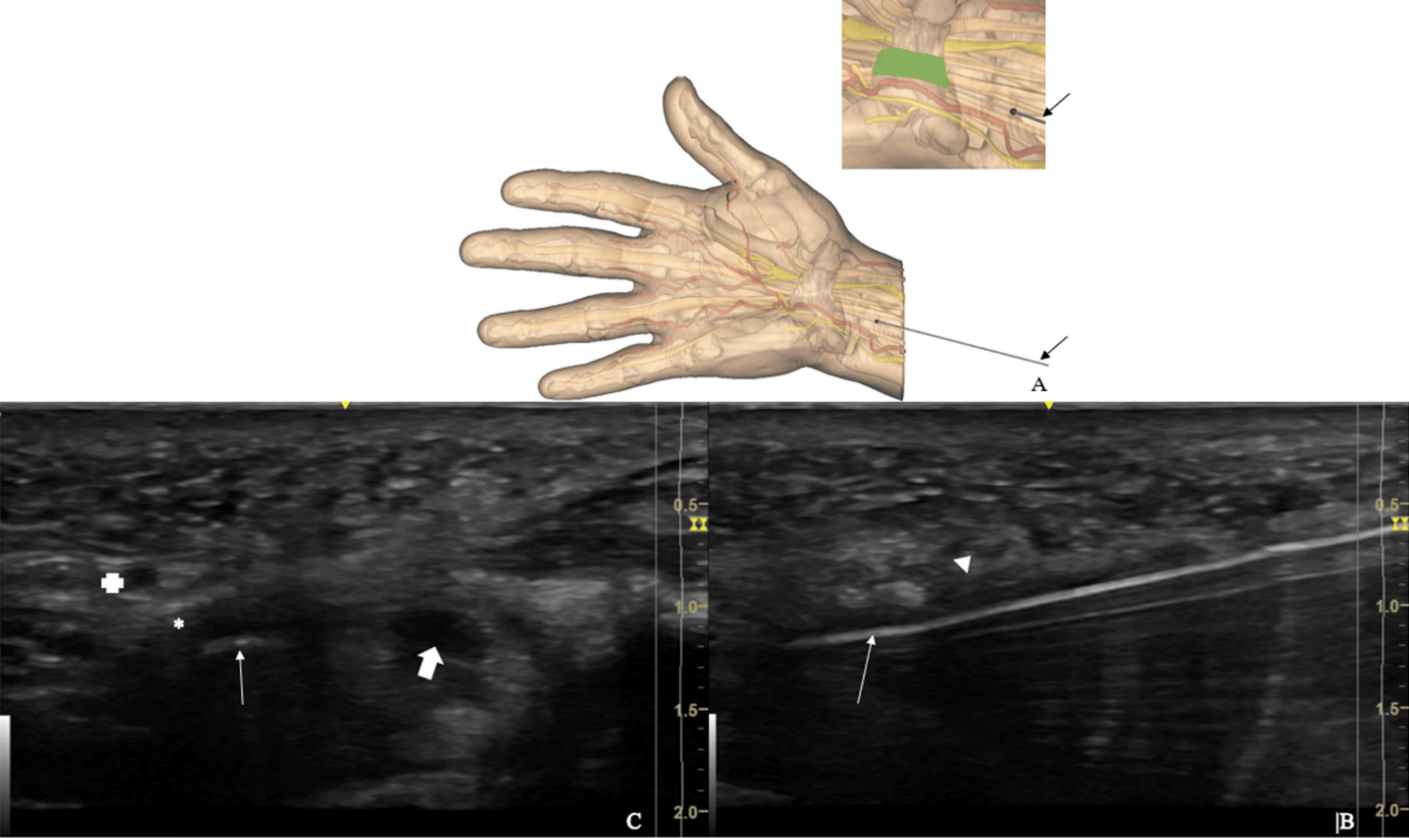
The round-ended stylus (arrow) is introduced through the entry point (red dot) in the carpal tunnel and defines the trajectory that the cutting instrument will follow in the safe zone between the hamate hook and the median nerve (green area) (A). Under sonography, the stylus (arrow) can be seen in the long-axis view under the TCL (arrowhead) (B). Short-axis view allows us to define the position of the future cut relative to the median nerve (solid arrow), the hamate hook (star) and the ulnar artery (cross) (C). This figure is the original work of the authors. TCL: Transverse carpal ligament

After retrieval of the catheter, a 1.5 mm rounded end stylus is introduced through the puncture by its rounded extremity, allowing visualization under sonography and palpation of the deep surface of the TCL, making some room for the instrument in the virtual space of the CT, and defining exactly the trajectory of sectioning (Figure 4A). The stylus is placed just lateral to the hook of the hamate, ensuring that it remains distant from the median nerve, the latter structure always radial to the instrument (Figure 4B). The physician makes sure that passing the stylus from the CT to the subcutaneous plane is not possible due to the interposed TCL, assessing the resistance of the ligament (Figure 4C). During this step, the sonography probe is placed alternately longitudinally and transversally, to obtain longitudinal and axial views, for perfect identification of the anatomical structures and position of the rounded end stylus. The stylus is then maintained exactly along the axis of the cut, and the sonography probe is aligned with the stylus. The sonography probe is then maintained locked in this position of the cutting pathway.

**Figure 5:**
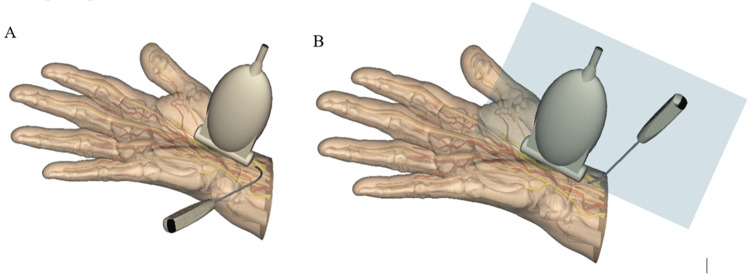
Introduction of the cutting instrument. In the first step, the instrument tip is obliquely oriented at 30° to 45° to the skin plane with its handle toward the surgeon (A). In the second step, the instrument is rotated around its tip’s axis to become vertical with its handle upside, the plan of the instrument rod curvature aligned with the probe plan (B). This figure is the original work of the authors.

**Figure 6:**
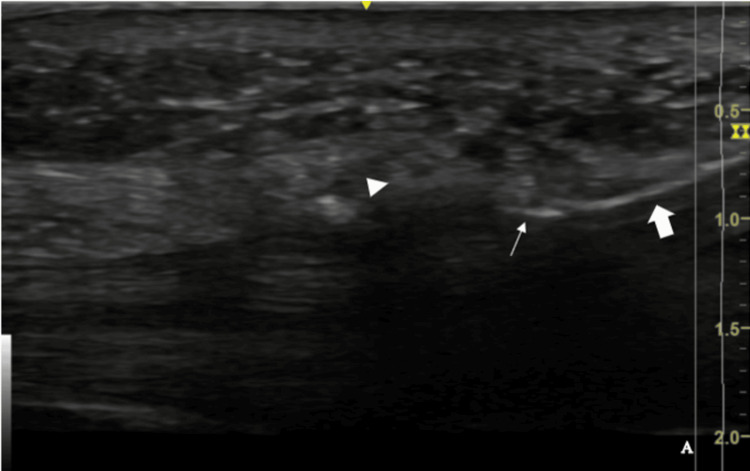
Sonographic long-axis view of the cutting instrument (solid arrow) in contact with the proximal part of the TCL (arrowhead). Note the hyperechoic line at the instrument tip (arrow). This line is generated by wave reflection over the flange located in the blade to show the cutting part of the instrument This figure is the original work of the authors. TCL: Transverse carpal ligament

After retrieval of the round-ended stylus, the Sono-Instrument is introduced through the entry point with initially the bevel of the instrument oriented at 30 to 45° to the skin plane, the handle of the instrument being located toward the surgeon (Figure 5A). Once the instrument tip is observed sonographically under the forearm aponeurosis, a 60° rotation of the instrument is performed around its tip’s axis to become vertical with its handle upside, the plane of the instrument rod curvature being aligned with the sonography probe plan (Figure 5B). This rotation turns the bevel of the instrument in the sagittal plane to be perpendicular to the TCL, allowing its release. The cutting part of the instrument can be seen under sonography as a hyperechoic line (Figure 6A). This image is generated by the ultrasound reflexion on a small flange built on the flat part of the instrument blade specifically to highlight its only sharp part. The cut is progressively performed under sonography, in the safe zone, by alternated oscillating movements in the sagittal plane, from proximal to distal, in an antegrade manner. The center of rotation of the alternating movements corresponds roughly to the skin entry point. Some cracking sounds may be heard during the release.

The section of the TCL is performed slightly obliquely, from dorsal to volar and medial to lateral. During the release, the probe can be moved in the long-axis view slightly ulnarly and radially alternately to confirm the position of the instrument relative to the hamate hook and median nerve, respectively. A 90° rotation of the sonography probe may also be performed to observe the relationship of these three structures in a short axis view. The distal border of the TCL is released with great care, because of the close vicinity of the superficial carpal arch and of the median motor and Berretini branches, by identifying accurately under sonography the instrument tip and these neurovascular structures.

After removal of the instrument, the rounded end stylus is re-introduced in the CT and is felt and seen displaced through the sectioned structure, up to the subcutaneous plane, without resistance. Should it not be the case, the Sono-Instrument is re-introduced, to selectively release the remaining uncut fibers of the TCL. No closure is needed, as there is no skin incision.

After the procedure, to prevent hematoma, tight compressing the site for a few minutes if there is blood oozing is beneficial. A compressive dressing is then applied, covering the entry point and the palm of the hand where the release was done. This dressing is removed a couple of hours after the procedure, and no more dressing is needed.

Patients are permitted to wash their hands and are encouraged to gradually resume daily activities, starting the day after the procedure. To reduce the risk of finger swelling and Complex Regional Pain Syndrome, wearing a sling for a few days to elevate the limb when not in use, along with active finger movement, is advised. Heavy labor is discouraged for several weeks to ensure proper ligament healing.

Surgery Precautions

Make sure that throughout the operation, the wrist remains in neutral alignment. If the patient moves his/her hand toward the wrist ulnar inclination during the procedure, it may endanger the distal part of the median nerve.

Intraoperative Difficulties

Occasionally, the ulnar pedicle may be radial to the hook of the hamate, limiting the safe zone. In such cases, it is recommended to incline the distal cut, to avoid injuring this structure. A marked angulation of the hook of the hamate may also limit visibility at the distal part of the CT.

If there is insufficient sonographic visualization of the anatomical structures, in case of anatomical abnormalities, or insufficient experience, consider converting to open release.

Postoperative Instructions

Patients should be informed that decreased gripping strength and pillar pain are common for weeks or months after the operation, like outcomes following open or endoscopic release. They are advised against forceful hand-gripping activities with the wrist in palmar flexion.

Tourniquet or Adrenalin Use:

The use of a tourniquet is not recommended. With a tourniquet, it is harder to identify the ulnar artery and the palmar arch under sonography. For the same reason, the use of adrenalin is not recommended during the procedure. Bleeding during the procedure doesn’t limit the sonographic visibility and could be information about procedure-related potential complications that should not be hidden by adrenalin or a tourniquet. In case of bleeding, five to ten minutes of compression is performed to prevent postoperative hematoma.

Expected outcomes

The first author of this paper (FM), the inventor, has now operated over 900 cases, without a single case of an iatrogenic lesion or CRPS. Patient satisfaction was very high. Moungondo et al. [16] conducted a prospective study to assess the safety and efficacy of Sono-Instruments® for percutaneous CT and trigger finger release. The study involved 30 patients and highlighted safety and efficacy. For the CTS group, 80% of working patients returned to their activities within a week. The device ensured effective, complication-free procedures, with significant DASH score improvements at two months post-operation [16]. None of the patients operated for CT release included in this study had been reoperated.

 The pin-hole method of sonography-guided CT release offers numerous benefits. For the surgeon, the operation is very quick. There is no need to use hand surgical instruments, other than the Sono-Instrument and a rounded end stylus. There is no need for a tourniquet, no need for an assistant. The technique is therefore particularly adapted to office surgery, under local anesthesia, reducing costs and inconveniences for patients. The technique is also very safe, given the design of the instrument and the fact that the surgeon cuts exactly where it is safe, not in the straight direction imposed by other types of cutting instruments. The instrument cuts specifically tense fibers and is quite inefficient on elastic fibers like nerves, vessels, or tendons. 

For the patient, percutaneous pin-hole surgery allows for an early return to professional and daily living activities. However, heavy professional work is not advisable due to unavoidable pain from ligament healing.

The major limitation is the necessity for the surgeon to learn hand sonography to master this new form of surgery. Training on cadaveric specimens and/or on-hand models is necessary before undertaking initial cases.

The absence of a visible scar might represent another drawback. Without appropriate documentation, it might be difficult for the patient to remember which side was operated on or even if a CT release has been performed. Good documentation in the medical chart is mandatory.

Complications

Iatrogenic injury to the neighboring nerves, vessels, and tendons, in addition to the incomplete release of TCL, might happen if the operating surgeon is not experienced with hand sonography. We did not encounter any complications with this technique. To decrease the risk of iatrogenic injuries, we advise the surgeons to undergo hand-based sonography training.

## Discussion

Several advantages can be pointed out for CT sonography-guided release when compared to traditional open release. Kamel et al. demonstrated in their study that patients who underwent CT sonography-guided release had significant improvement of their symptoms at two weeks postoperative, in addition to a high satisfaction rate, reaching 93% at one year [19]. Aguilo et al. in their multicenter study demonstrated the safety, efficacy, low complication rates, and favorable outcomes at one year postoperatively in patients treated with ultrasound-guided CT release using an Ultra Guide CTR device, although their technique was not purely percutaneous, it involved a 5 mm incision at the level of wrist crease [20]. One of the pros of CT sonography-guided release is rapid postoperative recovery and early return to activities of daily living and work [21]. Being purely percutaneous, our technique has a lower risk of infection and scar pain when compared to the open technique [1,22]. A comparative study published by Nakamichi et al. showed that patients who benefited from CT sonography-guided release with an incision smaller than 4mm had less scar pain and sensitivity when compared with patients who had an open or mini-open release of their TCL [7].

CT sonography-guided release can be of benefit for patients with certain medical conditions like diabetes, as it is well known that the diabetic population has a higher rate of infection, hypertrophic scarring, wound dehiscence, and scar contracture. Avoiding having an incision will decrease the risk of all these complications that can be morbid and affect the outcomes of surgery [23].

In terms of safety and efficacy, in echo of the study of Moungondo et al. [16], Petrover et al. conducted 129 percutaneous ultrasound-guided TCL releases using a hook knife, achieving a success rate of 100% measured by complete sectioning of the TCL and confirmed by magnetic resonance imaging one month after the procedure [24]. No complications were encountered intraoperatively, postoperatively, and up to six months of follow-up. Although some patients involved in their study had some anatomical variations of the median nerve, no iatrogenic injuries were encountered [4]. Various other studies also assessed the safety, risk of median nerve injury, and clinical outcomes of CT sonography-guided release, and all came in favor of CT sonography-guided release [8,10,25]. A systemic review done by Macdonald and Rea concluded that open CT release and endoscopic CT release are both associated with complications, where the rate of complications approached 4.1% and 5% respectively [26].

Several instruments designed for CT sonography-guided release were introduced to the market, like the MANOS device, manual hook knife [25], and the thread loop. The MANOS instrument needs two puncture sites, proximal and distal to the TCL, and the transection of the ligament depends on the appreciation of the surgeon or palpation rather than live monitoring using ultrasound [27]. The thread loop technique similarly needs two puncture sites, where the thread is looped around the TCL and pulled to transect the ligament, and likewise, the thread position cannot be controlled by ultrasound due to difficult visualization [28]. In contrast, the Sono-Instrument is designed in a way to be visible under ultrasound throughout the procedure; in addition, its cutting tip is outlined to be safe for the vital structures around the TCL like the median nerve and the flexor tendons. This convenience and utility make it ideal for office-based surgery [29].

## Conclusions

The authors have provided a detailed surgical technique, tips, and pitfalls of ultrasound-guided percutaneous CT release, a safe and effective pin-hole technique. Bilateral surgery is possible. The operation can be done with limited equipment and is adapted to office surgery. As there is no skin incision, the patient can go back to daily activities and light professional work the day after the operation. For the surgeon, mastering hand sonography is a prerequisite, and training is necessary.
